# Signatures of selection in *Mulinia lateralis* underpinning its rapid adaptation to laboratory conditions

**DOI:** 10.1111/eva.13657

**Published:** 2024-02-14

**Authors:** Zujing Yang, Ang Zhao, Mingxuan Teng, Moli Li, Hao Wang, Xuefeng Wang, Zhi Liu, Qifan Zeng, Liping Hu, Jingjie Hu, Zhenmin Bao, Xiaoting Huang

**Affiliations:** ^1^ MOE Key Laboratory of Marine Genetics and Breeding, College of Marine Life Sciences Ocean University of China Qingdao China; ^2^ Laboratory of Tropical Marine Germplasm Resources and Breeding Engineering Sanya Oceanographic Institution, Ocean University of China Sanya China; ^3^ Yantai Marine Economic Research Institute Yantai China; ^4^ Laboratory for Marine Fisheries Science and Food Production Processes, Qingdao National Laboratory for Marine Science and Technology Qingdao China

**Keywords:** adaptation, laboratory conditions, *Mulinia lateralis*, selection signature

## Abstract

The dwarf surf clam, *Mulinia lateralis*, is considered as a model species for bivalves because of its rapid growth and short generation time. Recently, successful breeding of this species for multiple generations in our laboratory revealed its acquisition of adaptive advantages during artificial breeding. In this study, 310 individuals from five different generations were genotyped with 22,196 single nucleotide polymorphisms (SNPs) with the aim of uncovering the genetic basis of their adaptation to laboratory conditions. Results revealed that *M. lateralis* consistently maintained high genetic diversity across generations, characterized by high observed heterozygosity (*H*_o_: 0.2733–0.2934) and low levels of inbreeding (*F*_is_: −0.0244–0.0261). Population analysis indicated low levels of genetic differentiation among generations of *M. lateralis* during artificial breeding (*F*_st_ <0.05). In total, 316 genomic regions exhibited divergent selection, with 168 regions under positive selection. Furthermore, 227 candidate genes were identified in the positive selection regions, which have functions including growth, stress resistance, and reproduction. Notably, certain selection signatures with significantly higher *F*_st_ value were detected in genes associated with male reproduction, such as *GAL3ST1*, *IFT88*, and *TSSK2*, which were significantly upregulated during artificial breeding. This suggests a potential role of sperm‐associated genes in the rapid evolutionary response of *M. lateralis* to selection in laboratory conditions. Overall, our findings highlight the phenotypic and genetic changes, as well as selection signatures, in *M. lateralis* during artificial breeding. This contributes to understanding their adaptation to laboratory conditions and underscores the potential for using this species to explore the adaptive evolution of bivalves.

## 1 INTRODUCTION

Unraveling the genetic basis of organisms' adaptation is essential for understanding how they acclimate to their environments (McKay & Latta, 2002; Storz, 2005). Experimental evolution, in particular laboratory selection, provides a method for studying adaptive responses in a controlled environment (Sandberg et al., 2017; Swamy & Zhou, 2019). Advances in our understanding of adaptive evolution have been driven by research using organisms amenable to laboratory conditions (LaCroix et al., 2017; Sandberg et al., 2017; Sterken et al., 2015). Typically, these studies focus on organisms with short generation times, such as *Saccharomyces cerevisiae* and *Drosophila melanogaster* (Claire et al., 2021; Sterken et al., 2015). For example, polymorphic populations or mixtures of various ancestral genotypes from one or more populations are utilized to establish experimental animal populations (Declerck et al., 2015; Graham et al., 2014). A key aspect of these studies is the application of selection pressure on organisms to enhance overall population adaptation (Sandberg et al., 2014; Vasi et al., 1994). In the case of *Escherichia coli*, Sandberg reported distinct genetically adaptive strategies in response to frequently switching growth substrates in the laboratory environment, allowing for continuous reproduction while maintaining exponential growth (Sandberg et al., 2017). Many approaches have been pursued to study adaptation (Coop et al., 2010; Tusso et al., 2021). In the rotifer *Brachionus plicatilis*, undergoing seven reproductive cycles under laboratory conditions, BayeScan (BS) analysis revealed 76 SNPs showing strong signals of selection, while genome‐wide association analysis (GWAS) further identified five SNPs associated with two key life‐history traits (Tarazona et al., 2019). This life history evolution, facilitating organisms' success in their environment, was linked to a variety of genes involved in diverse biological processes (Sandberg et al., 2017; Tarazona et al., 2019).

Mollusks represent one of the most ancient and evolutionarily successful groups of marine invertebrates, exhibiting a remarkable diversity in morphology, behavior, and lifestyle (Haszprunar & Wanninger, 2012; Hochner & Glanzman, 2016; Wanninger & Wollesen, 2019). The significant success of mollusks can be largely attributed to their wide range of adaptations to diverse environments, enabling the possibility of domestication or genetic improvement (Guo, 2021; Hedgecock, 2011; Ikhwanuddin & Abol‐Munafi, 2016). Currently, more than 30 mollusk species undergo varying degrees of domestication and are cultivated in hatcheries, encompassing numerous oysters, scallops, mussels, clams, and abalone species (Guo, 2021). The closure of the life cycle in hatchery production inevitably leads to genetic changes, including alterations in genes, genetic variation, and trait inheritance, whether through intentional selection or not (Gjedrem & Rye, 2018; Lowell, 2021). The genomes and transcriptomes of numerous mollusk species have been sequenced, providing rich information on the genetic and molecular mechanisms underlying mollusk development, function, immunity, and physiology, which has improved our understanding of mollusk genetics and adaptation (Abdelrahman et al., 2017; Guo, 2021).

The dwarf surf clam (*Mulinia lateralis*, 1822) is a small bivalve that is native to the western Atlantic Ocean from the Gulf of St. Lawrence to the Gulf of Mexico (Calabrese, 1969; Walker & Tenore, 1984). *M. lateralis* usually inhabits sandy and muddy sediments in estuarine and intertidal zones (Calabrese, 1969; Walker & Tenore, 1984). This species is a typical short‐generation species, reaching sexual maturity within two months (Calabrese, 1969). Sexually mature individuals are highly fecund, capable of laying up to 3 × 10^5^ eggs at a time, and can release gametes multiple times in a short period (Calabrese, 1970; Guo & Allen, 1994). Furthermore, induction spawning and artificial culture of *M. lateralis* can be easily achieved in the laboratory (Calabrese, 1969; Rhodes et al., 1975). These characteristics make *M. lateralis* a great potential model species for the study of bivalve biology and evolution. Recently, *M. lateralis* has been introduced into China, and we have systematically developed optimized culture conditions for this species in our laboratory (Yang et al., 2021, 2022). At present, after several generations of successful breeding, *M. lateralis* has clearly demonstrated its adaptability to laboratory conditions. Nonetheless, the genetic basis of *M. lateralis*' adaptation to laboratory conditions during artificial breeding is still unknown.

To this end, the present study aimed to investigate the phenotypic and genetic changes in *M. lateralis* during artificial breeding. Furthermore, the genomic selection signatures were scanned to identify regions associated with variations in adaptation to laboratory conditions during artificial breeding. Overall, our findings provide insights into the genetic basis of *M. lateralis*' adaptation to laboratory conditions during artificial breeding and reveal the potential of using this species to explore the adaptive evolution of bivalves.

## 2 MATERIALS AND METHODS

### 2.1 Clam culture, breeding, and collection

In 2017, a total of 200 *M. lateralis* individuals were introduced from the USA and then preserved in the MOE Key Laboratory of Marine Genetics and Breeding, Ocean University of China (Qingdao, China). The initial samples introduced were considered as the germplasm resource's first generation (G0). Adults from the G0 population were cultured to maturity in a recirculating system (temperature 21–22°C and salinity 27–28 psu). The seawater was pretreated with sand, activated carbon, and absorbent cotton, and then used as filtered seawater (FSW). A habitat substrate for *M. lateralis* consisted of a 3 cm layer of sand with particle sizes ranging from 0.25 to 0.5 mm. Some microalgal species, including *Isochrysis galbana*, *Nitzschia closterium*, *Chaetoceros muelleri*, *Platymonas helgolandica*, and *Chlorella pyrenoidesa*, were selected as the diet for *M. lateralis*, purchased from Jianyang Biological Technology Ltd. (Dalian, China). Feeding occurred three times a day, providing microalgae at a concentration of 5–10 × 10^4^ cells/mL^−1^, with a stocking density ranging from 600 to 800 individuals/m^2^.

**FIGURE 1:**
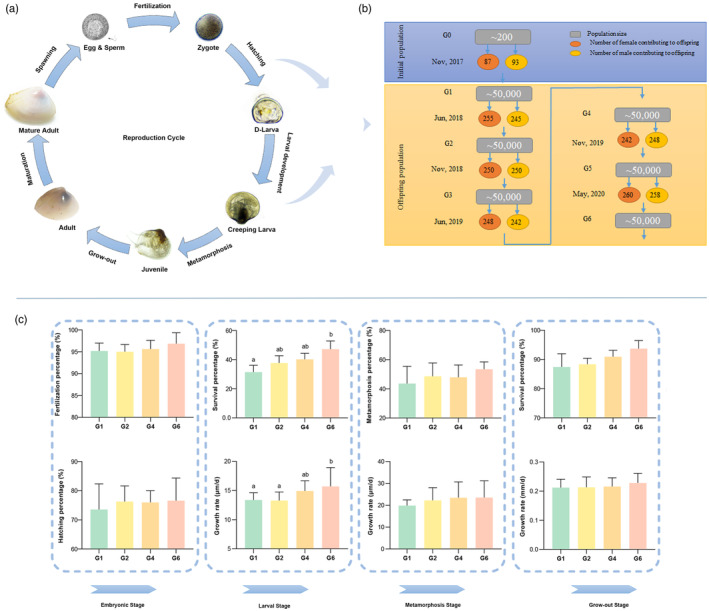
Reproduction cycle (a), breeding schedule (b), and phenotypic traits (c) of *Mulinia lateralis* populations. The gray square represents the population size of each generation. The ellipse represents the number of individuals contributing to the next generation (red for females and yellow for males). G0 refers to the initial population, while G1‐G6 refers to the offspring populations. Different colors indicate the *M. lateralis* population of different generations.

According to Figure 1a,b, mature individuals were selected as the foundation of the offspring population (G1), and each individual was independently subjected to thermal stimulation conditions for spawning induction. After collecting eggs from 87 females and sperm from 93 males, they were thoroughly mixed in a 3:1 ratio of sperm‐to‐egg to maximize the number of mature individuals contributing to offspring production. The culture of the offspring followed the methodology described in our previous studies (Yang et al., 2021, 2022). In brief, zygotes were transferred to 15‐L aquariums (30 cm × 25 cm × 20 cm) at a density of <50 eggs mL^−1^ and a temperature of 21–22°C for embryo incubation. After 14 h, zygotes rapidly developed into D‐shaped larvae, which were then collected and transferred to clean 15‐L aquariums filled with FSW. Thereafter, D‐shaped larvae progressed through stages including larval (1–10 days post‐fertilization (dpf)), metamorphosis (11–20 dpf), and grow‐out (21–50 dpf). In the meanwhile, adequate aeration of the aquariums ensured saturated oxygen concentration, and seawater was fully renewed every day with FSW at a temperature of 21–22°C. Aquariums were replaced every two days and sterilized with a 0.2% potassium permanganate solution. During this process, larvae were fed microalgae three times per day. At the larval stage, we initiated feeding *I. galbana* and gradually incorporated *P. helgolandica* at a concentration of 2.4 × 10^3^ cells/mL^−1^ during the D‐shaped stage, increasing to 12 × 10^3^ cells/mL^−1^ before the metamorphic stage. Larval stocking density remained less than 7 larvae/mL^−1^. At the metamorphic stage, the diet transitioned to a mixture of *I. galbana*, *P. helgolandica*, and *Nitzschia Closterium* (1:1:1) at a concentration of 3–4 × 10^4^ cells/mL^−1^, and the larvae were cultured at a stocking density of less than 5 larvae/mL^−1^. At the growth‐out stage, due to space constraints, some individuals were randomly selected and transferred to larger aquariums (1 m × 1 m × 0.5 m) with a 3 cm sandy substrate for benthic culture. They were fed a mixture of *C. muelleri* and *C. pyrenoidesa* (1:1) at a concentration of 5–10 × 10^4^ cells/mL^−1^, with a stocking density of 600–800 individuals/m^2^. Three aquarium replicates were conducted using a completely randomized design. Subsequently, adults from the G1 population were cultured to maturity for the next generation. This cycle allowed the successful establishment of offspring from different generations in our laboratory (Figure 1b). In the meanwhile, mature individuals from different generations were collected, and various tissues, including the gill, mantle, muscle, gonad, and foot, were quickly dissected and stored at −80°C for subsequent experiments.

### 2.2 Phenotypic trait statistics

Five traits, including fertilization percentage, hatching percentage, survival percentage, metamorphosis percentage, and growth rate, were calculated to evaluate the performance of different generations of *M. lateralis* throughout the life cycle (Figure 1c). For the embryonic stage, fertilization percentage was calculated as (the number of zygotes)/(the number of eggs) × 100%, and hatching percentage as (the number of D‐shaped larvae)/(the number of zygotes) × 100%. During the larval stage, survival percentage was determined by (the number of larvae at 10 dpf)/(the number of larvae at 1 dpf) × 100%, and the growth rate (μm/d) by (shell length of larvae at 10 dpf − shell length of larvae at 1 dpf)/10 d. At the metamorphosis stage, the metamorphosis percentage was calculated using (the number of juveniles at 20 dpf)/(the number of creeping larvae at 11 dpf) × 100%, and the growth rate (μm/d) as (shell length of juveniles at 20 dpf − shell length of creeping larvae at 11 dpf)/10 d. For the grow‐out stage, survival percentage was assessed by (the number of adults at 50 dpf)/(the number of juveniles at 21 dpf) × 100%, and the growth rate (mm/d) = (shell length of adults at 50 dpf − shell length of juveniles at 21 dpf)/30 d. Additionally, 30 larvae/juveniles/adults were measured to calculate the growth rate for each stage. These traits were calculated based on three replicates and presented as mean ± standard deviation (S.D.). Data were analyzed using IBM SPSS Statistics23 and depicted graphically using GraphPad8.0. A one‐way analysis of variance (ANOVA) followed by a post hoc comparison of means based on Tukey's test was performed to determine significant differences among generations (*p* < 0.05). Percentages were log_10_‐transformed to obtain normality and homogeneity of variance before ANOVA. The normality of data was confirmed by Kolmogorov–Smirnov's test and the homogeneity of variances by Levene's test.

### 2.3 DNA extraction and sequencing

Genomic DNA was extracted from the gills of *M. lateralis* by the TIANamp Marine Animals DNA Kit (No. DP324, TIANGEN, China) following the manufacturer's protocol. Subsequently, DNA was purified using the Column DNA purification kit (No. B610367, Sangon Biotech, China). The quality, purity, and concentration of DNA were assessed through electrophoresis on 1.5% agarose gels and NanoDrop Lite (Thermo Fisher, Waltham, MA, USA). IsoRAD sequencing libraries were then constructed using the method developed by Wang et al. (2016). Sequencing was performed on the Illumina Hiseq2000 platform (Novogene, China), generating 150 bp paired‐end reads for subsequent analyses.

### 2.4 Genotyping and SNP annotation

Applying three filtering steps, raw reads were processed by FastQC (https://github.com/s‐andrews/FastQC) to obtain high‐quality, clean reads. The filtering steps included: (i) removing reads with ambiguous basecalls (N); (ii) removing reads with long homopolymer regions (>10 bp); and (iii) removing reads with excessively low‐quality bases (>20% of bases with a quality score <10) (Andrews, 2010). RADtyping (https://github.com/jinzhuangdou/RADtyping), renowned for its general applicability to various forms of RAD‐seq data processing, was used to discover and genotype SNPs from the clean reads (Fu et al., 2013). In short, RAD tags were extracted from the genome of *M. lateralis*, serving as a reference based on the recognition sites of the BsaXI enzyme. The reference genome, a high‐quality chromosome‐level genome consisting of 19 chromosomes, was assembled using Illumina short‐reads, PacBio long‐reads, and Hi‐C scaffolding technology (unpublished). Clean reads were aligned to the reference using SOAP2 (https://github.com/ShujiaHuang/SOAPaligner) (Li et al., 2009), and SNPs were genotyped using the codom_calling.pl pipeline implemented in the RADtyping package with default parameters. Concurrently, RAD tags with coverage greater than 4× were used for genotyping, and an improved maximum likelihood (iML) algorithm was used to exclude repetitive sites from genotyping. Subsequently, the genotyped SNPs underwent processing using Plinkv1.9 (https://www.cog‐genomics.org/plink2/) with the following criteria: SNP call frequency (>0.9), minor allele frequency (>0.05), and individual call rate (>0.9) (Chang et al., 2015). Finally, the filtered SNPs were used for downstream analysis, and their annotation was performed using the SnpEff (https://pcingola.github.io/SnpEff/) with default parameters (Cingolani et al., 2012).

### 2.5 Genetic diversity, population structure, and linkage disequilibrium (LD)

Firstly, we evaluated the changes in genetic diversity of *M. lateralis* across different generations. Minor allele frequency (MAF), observed heterozygosity (*H*_o_), expected heterozygosity (*H*_e_), and inbreeding coefficient (*F*_is_) were calculated using Plink v1.9. Nucleotide diversity (π), fixation index (*F*_st_), and Tajima'D were evaluated through VCFtools (https://vcftools.github.io/) and averaged in 40 kb windows (Danecek et al., 2011). Secondly, the genetic structure of *M. lateralis* was assessed through principal component analysis (PCA) and a phylogenetic tree. PCA was conducted using GCTA (Yang et al., 2011), and the first two‐dimensional coordinates were plotted using the ggplot2 package (Villanueva & Chen, 2019). A neighbor‐joining (NJ) tree was constructed using Iqtree (Minh et al., 2020) with a bootstrap value of 1000, and the visualization was performed using iTOL (https://itol.embl.de/). In addition, linkage disequilibrium (LD) decay was analyzed by PopLDdecay with a maximum distance of 50 kb, and the average *r* ^2^ of pairwise markers was calculated (Zhang et al., 2019).

### 2.6 Selection signature analysis

To detect selection signatures in *M. lateralis* during artificial breeding, we scanned the genome using the ratio of θ_π_ and the fixation index (*F*_st_) between the initial population (G0) and offspring of advanced generation (G6). The estimation of θπ and *F*_st_ was conducted with sliding windows of 40 kb that had 20 kb overlap between adjacent windows. Subsequently, the ratio of θπ values at the same chromosomal location was computed. Regions under selection were identified using an empirical procedure (Li et al., 2018). Briefly, candidate genomic regions with significantly biased θπ ratios (top 5% largest and smallest) and significantly high *F*_st_ values (top 5% largest) of the empirical distribution were regarded as regions with strong selection signatures across the genome. Among them, regions with significantly biased θπ ratios (top 5% largest) and significantly high *F*_st_ values (top 5% largest) were categorized as exhibiting strong positive selection signatures, whereas regions with significantly biased θπ ratios (top 5% smallest) and significantly high *F*_st_ values (top 5% largest) were defined as regions with strong negative selection signals. The statistical significance was determined using permutation tests through random replacement of samples in population pairs (*p* < 0.05).

### 2.7 Candidate genes and functional analysis

According to genome annotation, candidate genes under selection were defined as within or overlapped with regions exhibiting selection signatures. Transcriptomic data generated from gonad tissues collected from the G0 and G6 generations from the same population used in the genetic analysis were used to explore the expression pattern of candidate genes associated with reproduction during artificial breeding. Specifically, sexually mature individuals, including males and females, were randomly selected from both generations and cultured under identical laboratory conditions. Total RNA was isolated based on the conventional guanidinium isothiocyanate method (Hu et al., 2006). Five RNA samples from each sex per generation were used for transcriptome construction. RNA sequencing libraries were constructed using an RNA‐seq library kit (Vazyme, China), and RNA‐seq data was generated using the high‐throughput Illumina HiSeq2000 platform. After quality control, high‐quality reads were mapped to the *M. lateralis* genome using STAR (Dobin et al., 2013), and the expression level was calculated in transcripts per million (TPM) using the RNA‐Seq by Expectation Maximization (RSEM) method. Meanwhile, the transcriptome data for G6 has been previously published (PRJNA862073; Li et al., 2022), while the transcriptome data of the G0 generation will be released with the genome article (as shown in Supplementary Sheet 6). Three transcriptome samples from each sex per generation were used for differential expression analysis. The expression profiles of candidate genes were normalized and represented as relative expression (TPM_candidate genes_/TPM_EF1A_), with elongation factor 1A (EF1A) serving as an internal reference gene widely used in bivalve (Volland et al., 2017; Zhao et al., 2018). Significant differences between G0 and G6 generations were determined using independent‐sample t‐tests (*p* < 0.05, *n* = 3).

To gain insights into the biological significance of selection signatures, we performed gene ontology (GO) and Kyoto Encyclopedia of Genes and Genomes (KEGG) functional enrichment analysis using OmicShare (https://www.omicshare.com/tools/). GO terms and KEGG pathways with *p*‐values less than 0.05 were considered significantly enriched.

## 3 RESULTS

### 3.1 Phenotypic traits of *M. lateralis*

As shown in Figure 1c, the phenotypic traits of *M. lateralis* revealed the performance of different generations throughout the life cycle. During the embryonic stage, the fertilization and hatching percentages of *M. lateralis* exhibited a tendency to increase with successive laboratory reproductions, with fertilization percentages ranging from 95.18 ± 1.47% to 96.86 ± 2.03% and hatching percentages ranging from 73.54 ± 7.88% to 76.61 ± 6.73%. The ANOVA test revealed no significant differences between generations (*p* > 0.05). At the larval stage, significant differences were observed in larval survival and growth among different generations (*p* < 0.05), with the advanced generation (G6) displaying the highest survival percentage (47.22 ± 4.81%) and growth rate (15.71 ± 3.13 μm/d), respectively. Similar trends were observed in the metamorphosis and grow‐out stages of *M. lateralis*, with the advanced‐generation (G6) exhibiting the highest metamorphosis percentages (53.46 ± 4.57%) and survival percentages (93.72 ± 2.26%). In addition, high growth rates were found in the metamorphosis (19.84 ± 2.56–23.54 ± 7.58 μm/d) and grow‐out stages (0.21 ± 0.03–0.23 ± 0.03 mm/d) across different generations, but no significant differences were detected. Overall, *M. lateralis* demonstrated the ability to maintain stable phenotypic traits during artificial breeding in the laboratory.

### 3.2 Sequencing and genotyping of *M. lateralis*

After the sequencing of libraries, a total of 821,047,756 raw reads were generated from 310 samples (Supplementary sheet 1). After quality filtering, 804,926,229 clean reads were retained, with an average effective mapping rate of 98.85%. The data showed high quality scores, with averages of 97.57% and 94.06% for Q20 and Q30, respectively. The GC content was stable, ranging from 45.37% to 48.03%. On average, 33.75% of reads containing RAD tags were uniquely mapped to the reference genome of *M. lateralis*, reaching an average depth of approximately 68× per sample (Supplementary sheet 2). In total, 34,901 SNPs were genotyped in these samples. After filtering, 22,196 SNPs from 280 samples, including G0 (44), G1 (60), G2 (54), G4 (53), and G6 (69), were retained for subsequent analysis.

The distribution of SNPs on *M. lateralis* chromosomes is illustrated in Figure S1a, with an average of 1169 SNPs distributed on each chromosome. The largest number of SNPs was observed on Chr1 (1894), while Chr19 had 324 SNPs (Figure S1b). Among all SNPs, 36.28% were located in intergenic regions, 35.23% in intragenic regions (24.42% in exons and 10.81% in introns), and 0.61% in UTR regions (0.22% in 5’ UTR and 0.39% in 3’ UTR) (Table S1). Of the different types of mutations, transitions (55.46%) were more frequent than transversions (44.54%), resulting in a transitions/transversions ratio of 1.25. Specially, the percentages of A/G and C/T transitions were very similar (27.56% and 27.9%, respectively), while the percentages due to A/T, A/C, G/T, and G/C transversions showed significant differences (20.98%, 9.57%, 9.76%, and 4.23%, respectively) (Table S2).

### 3.3 Genetic changes of *M. lateralis* during artificial breeding

**FIGURE 2:**
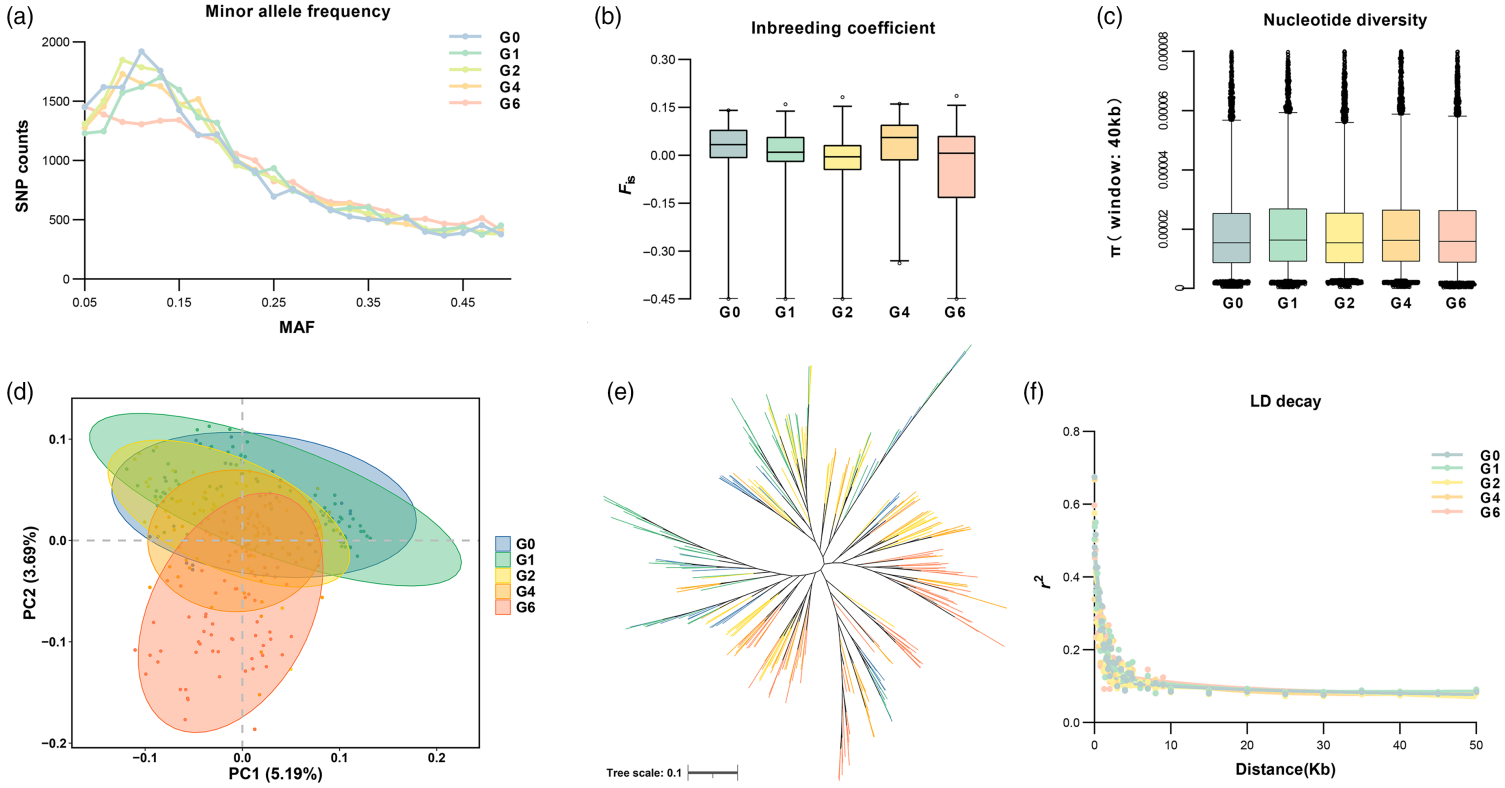
Genetic parameters of the initial population (G0) and offspring of different generations (G1–G6) in *Mulinia lateralis*. (a) Distribution of minor allele frequency (MAF); (b,c) Inbreeding coefficient and nucleotide diversity (40 kb windows); (d) Principal component analysis (PCA); (e) Neighbor‐joining tree; (f) Linkage disequilibrium decay (LD decay). Different colors indicate the *M. lateralis* population of different generations.

The dataset comprising 22,196 SNPs was used to estimate the genetic diversity of *M. lateralis* (Table 1). MAF exhibited variability among generations, with mean MAF ranging from 0.1922 (G0) to 0.2011 (G6). Examining the distribution of MAF (Figure 2a) revealed a lower proportion of rare SNPs (0.05 < MAF <0.1) in the G6 generation (16.45%) compared to the G0 generation (18.12%). Across generations, average *H*_o_ ranged from 0.2733 to 0.2934, while mean *H*_e_ ranged from 0.2842 to 0.2908 (Table 1). Notably, the *H*_o_ and *H*_e_ values of the G1, G2, and G6 populations were nearly identical (0.01 difference), while the G0 and G4 populations exhibited relatively larger differences between *H*_o_ and *H*_e_ values (0.0168 and 0.0138), respectively. Patterns revealed by heterozygosity analysis were supported by inbreeding analysis (Figure 2b, Table 1). *F*_is_ values varied from −0.0244 to 0.0261 for each generation, with the G0 and G4 populations showing higher *F*_is_ values of 0.0128 and 0.0261, respectively. As shown in Figure 2c and Table 1, all generations exhibited equal levels of π, with mean values ranging from 1.91 × 10^−5^ to 2.01 × 10^−5^. In summary, *M. lateralis* consistently maintained high levels of genetic diversity during artificial breeding.

**TABLE 1:** Genetic diversity indices among different generations of *Mulinia lateralis* assessed by 22,196 genome‐wide SNPs.

Generation	*n*	MAF	*H*_o_	*H*_e_	*F*_IS_	π
G0	44	0.1922	0.2733	0.2901	0.0128	1.91E‐5
G1	60	0.1986	0.2871	0.2908	−0.0076	2.01E‐5
G2	54	0.1975	0.2883	0.2842	−0.0146	1.91E‐5
G4	53	0.1976	0.2768	0.2906	0.0261	2.00E‐5
G6	69	0.2011	0.2934	0.2869	−0.0244	1.96E‐5

Abbreviations: *F*_IS_, mean inbreeding coefficient; *H*_e_, mean expected heterozygosity; *H*_o_, average observed heterozygosity; MAF, average minor allele frequency; n, number of samples; π, mean nucleotide diversity.

To explore the genetic relationships among different generations of *M. lateralis*, we conducted principal component analysis (PCA) and generated a neighbor‐joining phylogenetic tree. The PCA results depicted different clusters for the initial population and the offspring of different generations (Figure 2d). Principal Component 1 (PC1) explained 5.19% of the total variance, separating G0, G1, and G2 from other generations, while PC2 explained 3.69% of the total variance, describing the genetic differentiation of G6 from other generations. Notably, all generations of *M. lateralis* were collectively clustered in the same class without substantial separation. The NJ tree revealed that *M. lateralis* individuals from different generations clustered into multiple subclades, indicating close genetic relatedness and a common ancestry (Figure 2e). Tajima's D is a widely used metric for inferring the types of variations in DNA sequences. As illustrated in Figure S3, Tajima's *D* values for *M. lateralis* of different generations generally exceeded 0, with respective average values of 0.834, 1.097, 1.042, 1.034, and 1.139, suggesting that *M. lateralis* was under selection. Simultaneously, an interesting finding reveals that 258 regions exhibit Tajima's *D* values exceeding 0 in the G0 generation, whereas these values drop below 0 in the G6 generation (Figure S4). Nevertheless, the quantity of these regions is notably less than those with values exceeding 0.

Pairwise *F*_st_ indices between generations of *M. lateralis* were estimated to infer the genetic distance between generations. The levels of genetic differentiation among generations were low, with average *F*_st_ values of 0.0192 (Table 2). The highest genetic distance (0.0371) was observed between G6 and the earlier generations (G1), while the lowest genetic distance (0.0054) was observed between G1 and G0, respectively. The average *r* ^2^ values of LD in different generations decreased with increasing marker distance between pairwise SNPs, showing a rapidly declining trend with the first 50 kb (Figure 2f). Overall, genetic differentiation did occur in *M. lateralis* during artificial breeding, but it remained at a relatively low level.

**TABLE 2:** The pairwise *F*_st_ among the different generations of *Mulinia lateralis*.

Generation	G1	G2	G4	G6
G0	0.0054	0.0118	0.0145	0.0319
G1	–	0.0145	0.0219	0.0371
G2	–	–	0.0122	0.0259
G4	–	–	–	0.0167

### 3.4 Detection of selection signatures involved in adaptation to laboratory conditions during artificial breeding

**FIGURE 3:**
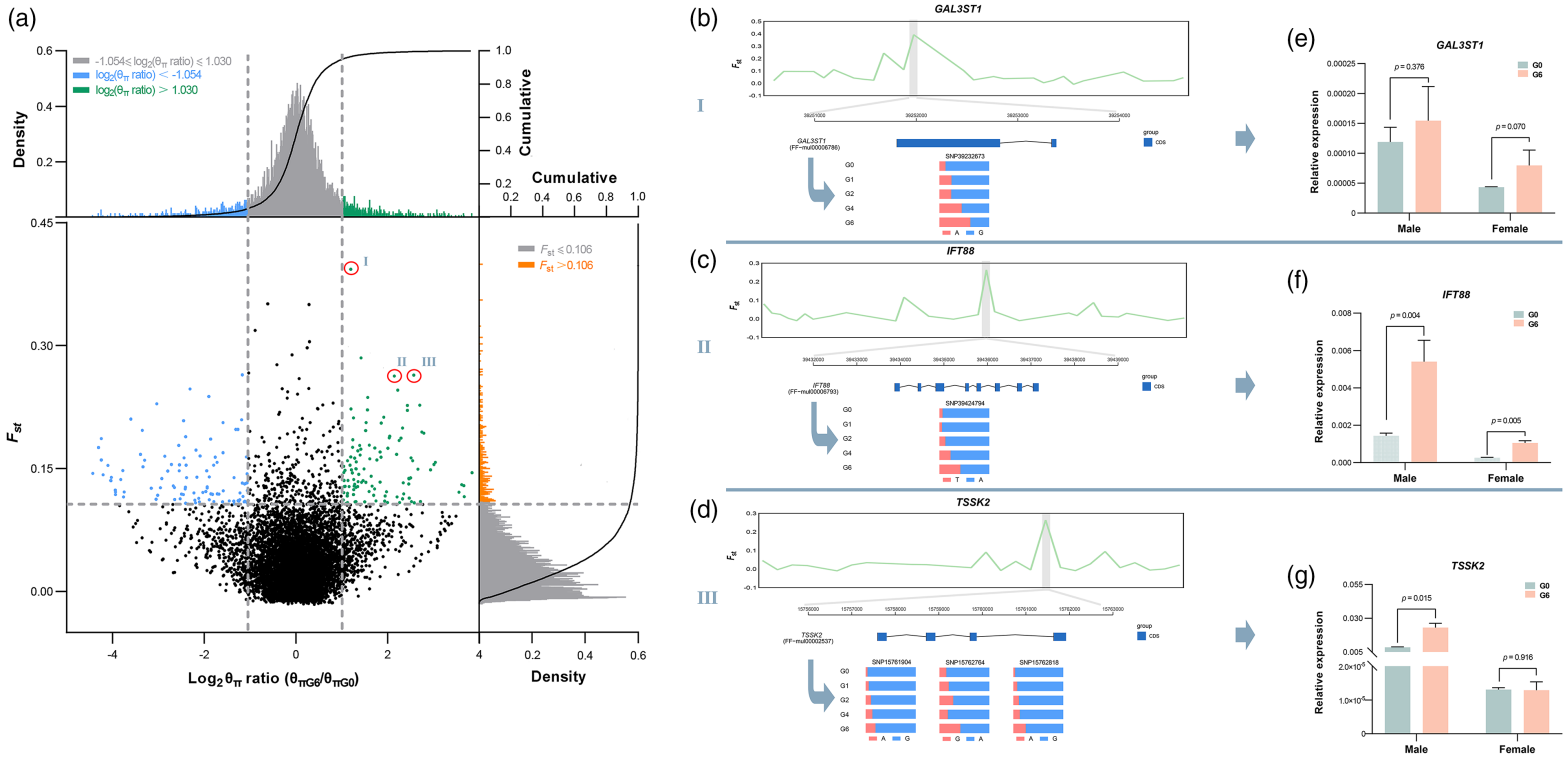
Identification of the genomic regions with selection signatures as well as candidate genes in *Mulinia lateralis* during artificial breeding. (a) Distribution of *F*_st_ values and log_2_ (θπ ratio) calculated in 40‐kb sliding windows with 20‐kb overlap between the initial population (G0) and offspring of advanced‐generation (G6). The blue data represent genomic regions under negative selection, and the green data represent genomic regions under positive selection. (b–d) Genetic characterization of *GAL3ST1*, *IFT88*, and *TSSK2* during artificial breeding, including genetic differentiation represented by the pairwise *F*_st_ value between G0 and G6 generations and genetic variation represented by allele frequencies of different generations. (e–g) Expression levels of *GAL3ST1*, *IFT88*, and *TSSK2* in gonad tissues of G0 and G6 generations. *p* indicates a significant difference between G0 and G6 generations, determined using independent‐sample *t*‐tests.

To detect the genomic signature of selection during artificial breeding, we scanned the genome of offspring by comparing the advanced generation (G6) with the initial population (G0). Using the top 5% cutoffs for the θπ ratio and *F*_st_ values (log_2_(θπ ratio) > 1.030 or log_2_θπ ratio < −1.054 and *F*_st_ >0.106), we identified 168 candidate regions under positive selection and 148 candidate regions under negative selection (Figure 3a, Supplementary sheet 3). In addition, these selected regions were unevenly distributed in the genome of *M. lateralis*, with chromosome 3 harboring more regions with selection signatures (48), including the region with the highest *F*_st_ value (0.3929) (Figure S2, Supplementary sheet 3).

Furthermore, 227 candidate genes were identified in positive selection regions, while 196 candidate genes were identified in negative selection regions (Supplementary sheet 3). Several important genes were identified in these regions, including *GAL3ST1*, *IFT88*, *TSSK2*, *MED1*, *CDC123*, *MKS1*, *THADA*, *DNAJB5*, *DNAH2*, and *CES5A*, which functionally involve a variety of biological processes. Of particular interest is the candidate gene (*GAL3ST1*) located in the region with the highest *F*_st_ value (*F*_st_ = 0.3929) (Figure 3b), which was associated with spermatogenesis during reproduction. Similar phenomena were also observed in male reproduction‐related genes (*IFT88* and *TSSK2*), which were identified in regions with high *F*_st_ values (Figure 3c,d). Moreover, we found that the variation in allele frequencies of these genes was a continuous, long‐term process during artificial breeding and eventually exhibited highly positive‐selected signatures in these regions. Transcriptomic data indicated that the expression of these selected genes was affected during artificial breeding, as evidenced by significant changes in expression levels. As shown in Figure 3e–g, *GAL3ST1*, *IFT88*, and *TSSK2* were highly expressed in gonad tissues of the advanced generation compared to the initial population (*p* < 0.05).

**FIGURE 4:**
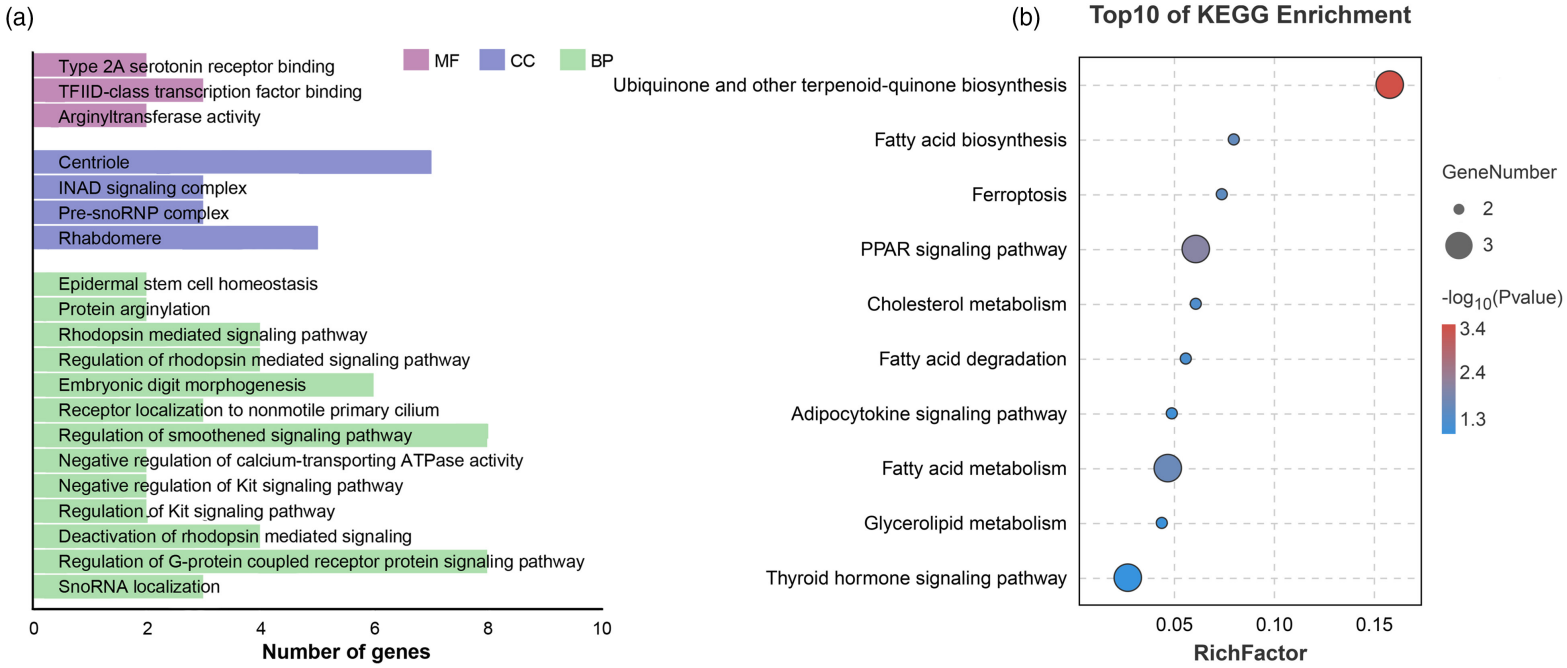
GO terms (a) and KEGG pathways (b) analysis of positively selected genes in *Mulinia lateralis*.

To further reveal the genetic basis of the adaptation of *M. lateralis*, positively selected genes were annotated in multiple categories of GO (Figure 4a, Supplementary sheet 4). The categories most significant in the “biological process” principal category were “SnoRNA localization” (3 genes), followed by “Regulation of G‐protein coupled receptor protein signaling pathway” (8 genes). In the principal category of “cellular component”, the two most significant categories were “Rhabdomere” (5 genes) and “Pre‐snoRNP complex” (3 genes). As for the “molecular function” principal category, the most significant category was “Arginyltransferase activity” (2 genes). In addition, these genes were mapped to the canonical reference pathways in the KEGG database. The top three enriched pathways were “Ubiquinone and other terpenoid‐quinone biosynthesis” (3 genes, *p* = 0.00036), “PPAR signaling pathway” (3 genes, *p* = 0.00589), and “Fatty acid metabolism” (3 genes, *p* = 0.01232) (Figure 4b, Supplementary sheet 5).

## 4 DISCUSSION

In the present study, all generations of *M. lateralis* were cultured under similar laboratory conditions, aiming to minimize environmental differences that could drive adaptive change between generations. Overall, we achieved a complete life cycle culture of the clam in the laboratory and maintained high reproductive performance during artificial breeding. This also confirms the potential of *M. lateralis* as a model bivalve, which is consistent with previous reports (Calabrese, 1969; Rhodes et al., 1975). Simultaneously, adaptive advantages in phenotypic traits were observed in subsequent *M. lateralis* generations, such as gradual increases in fertilization and larval survival, providing evidence of the species' adaptation to laboratory conditions.

In general, higher genetic variability within an experimental population enhances the likelihood of individuals possessing alleles better adapted to the environment, enabling them to survive, transmit favorable genetic characteristics to their offspring, and gradually undergo adaptive evolution (D'ambrosio et al., 2019; Durland et al., 2021). However, for experimental populations reared under laboratory conditions, artificial breeding usually contributes to a significant reduction in genetic variability, posing a threat to long‐term genetic progress as well as diminishing the adaptive capacities of populations (Felsenstein, 1965). For example, in a study on the drone fly (*Eristalis tenax* L.), the fourth and eighth generations of laboratory populations showed a severe lack of genetic diversity compared to natural populations, accompanied by phenotypic divergence (i.e. wing traits and abdominal color patterns) (Francuski et al., 2014). In contrast, we did not observe a similar phenomenon in the artificial breeding of *M. lateralis*, as offspring consistently maintained high genetic diversity. In a previous study, the loss of genetic variability in laboratory organisms was primarily due to the founder effect, followed by inbreeding (Francuski et al., 2014; Vlachos & Kofler, 2019). In our study, a strategic approach was employed by selecting more individuals from the germplasm population of *M. lateralis* as the initial population. This deliberate action aimed to increase the number of founders, thereby expanding the pool of genetic variations and overcoming the founder effect to some extent. In addition, during the artificial breeding of *M. lateralis*, offspring were generated from the initial population using random mating, without the imposition of deliberate artificial selection for certain traits. This approach effectively decreased the likelihood of inbreeding, a conclusion supported by the observed high heterozygosity and low levels of inbreeding across different generations of *M. lateralis*.

Nonetheless, the genetic structure of *M. lateralis* during artificial breeding exhibited dynamism rather than static constancy. Wherein, PCA analysis showed no substantial separation among the offspring of *M. lateralis*, yet there was a discernible tendency for the offspring of advanced generation to gradually deviate. This observed deviation might be attributed to selection, which often occurs in shellfish during artificial breeding (Guo et al., 2018; Langdon et al., 2003; Plough, 2012). In our study, Tajima's D results supported this hypothesis, as the values for *M. lateralis* of different generations generally exceeded 0. Selection, being one of the driving forces, plays an important role in the adaptive evolution of organisms. Individuals with certain genotypes exhibit great adaptability to their given environment, and their offspring show better population reproduction under natural selection, thereby influencing the overall genetic structure of the population (Burny et al., 2021; Langmüller et al., 2021; Neher, 2013). Genetic drift is also one of significant driving forces behind evolution, leading to the rapid reduction of genetic diversity in small populations and thereby swiftly altering their genetic structure (Lynch et al., 2016; Masel, 2011). *M. lateralis* was initially introduced to our laboratory from the USA with a relatively small initial population. Subsequently, it underwent long‐term cultivation in closed conditions, forming a relatively closed and small population. Furthermore, owing to the prevalent “sweepstakes effects” observed in bivalves (Hedgecock, 2011), the genetic drift observed in *M. lateralis* is entirely plausible, further confirming the results obtained from the PCA. Simultaneously, we noted consistently low genetic differentiation between generations of *M. lateralis* (*F*_st_ <0.05), and the genetic diversity of the offspring exhibited no significant decline. The marginal loss of genetic diversity and the minor fluctuations in *F*_st_ values across populations imply that, in comparison to selection, genetic drift played a relatively minor role (Furlan et al., 2012; Matos et al., 2015; Tarazona et al., 2019). In addition, we also observed an interesting finding that 258 regions exhibited Tajima's *D* values exceeding 0 in the G0 generation, whereas these values dropped below 0 in the G6 generation. Nevertheless, the quantity of these regions was notably less than those with values exceeding 0, indicating the presence of genetic drift in the offspring, but at a weak level. Hence, both selection and genetic drift are pivotal in the adaptation of *M. lateralis* to laboratory conditions, with selection exerting a predominant influence. In summary, genetic differentiation arising from both selection and drift occurred in the artificial breeding process of *M. lateralis*, though at a relatively modest level.

Although *M. lateralis* populations did not exhibit obvious genetic differentiation among generations at the level of the entire genome (*F*_st_ <0.05), significant selection signatures were observed in specific genomic regions. These findings were not unexpected, considering the consistent imposition of laboratory conditions throughout the breeding process of *M. lateralis* populations. Laboratory adaptation is an umbrella term for conditions that can be attributed to experimental evolution (Matos et al., 2002; Simões et al., 2007). Examples of such factors include adaptation to high stocking density (Hoffmann et al., 2001; Plough et al., 2014), sexual selection (Fricke & Arnqvist, 2007; Gjedrem & Rye, 2018), temperature (Evans & Langdon, 2006; McCarty et al., 2022), and adaptation to laboratory diet sources (McFarland et al., 2020; Sandberg et al., 2017). Genomic signatures of adaptation through experimental evolution have been identified in many species, such as *B. plicatilis* (Tarazona et al., 2019), *Daphnia magna* (Orsini et al., 2012), and *D. melanogaster* (Claire et al., 2021). For instance, in the case of *B. plicatilis*, researchers reported genomic signatures indicating rapid phenotypic divergence in laboratory populations, supporting the evolution of divergent life‐history traits over a short time span in rotifer populations (Tarazona et al., 2019). Nonetheless, the genetic variants crucial for adaptation to different environments are not fully shared between these studies. To the best of our knowledge, recent studies involving bivalves have identified specific genomic signatures of environmental adaptations, with limited focus on laboratory cultivation (Abdelrahman et al., 2017; Durland et al., 2021; Hornick & Plough, 2019; Li et al., 2021; Lim et al., 2021; Scobeyeva et al., 2021; Zhong et al., 2016). In the present study, 316 candidate regions were identified in the offspring of advanced generation under selection using the top 5% cutoffs of the θπ ratio and *F*_st_ values, of which 168 showed significant positive selection. Considering that these genomic signatures enable this species to adapt to laboratory culture, our findings can serve as a foundational platform for in‐depth studies concentrating on bivalve adaptation.

Adaptation is a complex process that helps organisms thrive in an environment and is often associated with various biological processes. As organisms adapt to specific environments, their growth and metabolism typically undergo great changes due to variations in environmental conditions (Teletchea, 2016). In the present study, several genes associated with growth and metabolism, such as *EGF1*, *MED1*, *PHYH*, and *CDC123*, exhibited significant positive selection signatures in *M. lateralis* during artificial breeding. Furthermore, several immune‐related genes, including *BIRC2*, *CDC20*, *MKS1*, etc., showed strong signals of positive selection. Generally, experimental organisms are cultured in laboratory conditions, subjected to the constraints of limited spaces and high density. These conditions frequently exert pressure on their immune response and may even lead to the occurrence of large‐scale diseases (Lin et al., 2018). Fortunately, during artificial breeding, *M. lateralis* had high survival rates without outbreaks of disease.

Improving reproduction in a breeding population has been recognized as a key factor for optimizing offspring production (Fox & Czesak, 2000; Izquierdo et al., 2001; Lind et al., 2019). In our study, we identified significant selection signatures in the genomic regions of *M. lateralis* associated with reproduction, especially in the regions of male reproduction‐related genes (such as *GAL3ST1*, *IFT88*, and *TSSK2*), showing higher *F*_st_ values. Of particular interest is the candidate gene (*GAL3ST1*) located in the region with the highest *F*_st_ value (*F*_st_ = 0.3929), which encodes a protein known to be involved in spermatogenesis (Suzuki et al., 2010). *IFT88* and *TSSK2* are genes associated with spermatogenesis (Yap et al., 2022; Zhang et al., 2020). Furthermore, we observed an increase in the expression of *GAL3ST1*, *IFT88*, and *TSSK2* during breeding in gonad tissues of advanced‐generation animals compared to the initial population (*p* < 0.05). In addition, the allele frequency in these genes showed gradual changes between generations with artificial breeding. Besides, we also significantly identified several genomic signatures that are localized to functional genes (e.g. *DNAH2* and *CES5A*) responsible for determining sperm morphology and capacitation. Specifically, *DNAH2* is a gene associated with various morphological abnormalities of sperm flagella (Li et al., 2019), and *CES5A* is required for sperm capacitation and male fertility (Ru et al., 2015). Previous studies have shown that male traits typically exhibit substantial additive genetic variance and rapid evolutionary responses to selection (Evans & Simmons, 2008), which have been widely confirmed in vertebrates, such as humans (Swanson & Vacquier, 2002), mice (Vicens et al., 2014), and birds (Kleven et al., 2009). Consequently, we speculated that significant selection signatures in male genomic regions of *M. lateralis* may be related to the rapid selective response in sperm‐associated genes during laboratory breeding. One possible explanation for this phenomenon is that males with the most abundant and active sperm may be more successful reproductively during mass spawning of *M. lateralis*, where sperm‐associated genes play a key functional role in regulating these processes.

The field of experimental evolution is expanding, with an increasing number of model organisms amenable to experimental culture and manipulation in a laboratory environment. In the present study, we have provided a detailed elucidation of the genetic basis of adaptation of *M. lateralis* to laboratory conditions during artificial breeding based on the long‐term breeding practices of this species. Our findings exhibit the potential of using *M. lateralis* as a valuable model for exploring the adaptive evolution of bivalves. However, laboratory conditions are defined by a number of various and interacting factors, including temperature, stocking density, diet, etc. In the present study, it proves challenging to distinguish the individual effect of each factor, hindering our ability to delve into the specific mechanisms underlying adaptation to any particular environmental condition. Thus, further research is needed to gain deeper insights into the adaptations of *M. lateralis* to laboratory conditions in the future breeding program.

## 5 CONCLUSION

This study reports the phenotypic and genetic changes as well as selection signatures of *M. lateralis* populations of different generations reared under laboratory conditions. The results demonstrate that *M. lateralis* could consistently maintain high genetic diversity during artificial breeding, with relatively low levels of genetic differentiation among generations. Several genomic regions showing significant selection signatures were identified, harboring genes associated with key adaptive traits throughout the life cycle of *M. lateralis*. Overall, our findings provide insights into the genetic basis for adaptation of *M. lateralis* to laboratory culture during artificial breeding and reveal the potential of using this species to explore the adaptive evolution of bivalves.

## FUNDING INFORMATION

The project was supported by Key R&D Project of Shandong Province (2021ZLGX03), Sanya Yazhou Bay Science and Technology City (SKJC‐KJ‐2019KY01), Earmarked Fund for Modern Agro‐industry Technology Research System (CARS‐49), Yantai Science and Technology Project (2020MSGY063) and National Infrastructure of Fishery Germplasm Resources (2019DKA30470).

## CONFLICT OF INTEREST STATEMENT

The authors have no conflict of interest to declare.

## Supporting information


**Figure S1.** Distribution of single nucleotide polymorphisms (SNPs) in the genome of *Mulinia lateralis*. (a) Locations and density of SNPs; (b) Numbers of SNPs in different chromosomes.

**Figure S2.** Distribution of selected regions in *Mulinia lateralis*. The green represents positively selected regions, while the blue represents negatively selected regions.

**Figure S3.** Tajima’s *D* values in *Mulinia lateralis* of different generations.

**Figure S4.** Distribution of Tajima’s *D* values in genomic regions of *Mulinia lateralis*. The green represents the G0 generation, while the purple represents the G6 generation.

**Table S1.** Characteristics and numbers of SNPs in *Mulinia lateralis* populations.

**Table S2.** Summary of nucleotide substitutions in *Mulinia lateralis* populations.

**Supplementary sheet 1.** Statistics of sequencing libraries of *Mulinia lateralis*.

**Supplementary sheet 2.** Summary of data processing in *Mulinia lateralis* libraries.

**Supplementary sheet 3.** List of the genomic regions with selection signatures as well as candidate genes in *Mulinia lateralis*.

**Supplementary sheet 4.** GO terms of the selected genes in *Mulinia lateralis*.

**Supplementary sheet 5.** KEGG pathways of the selected genes in *Mulinia lateralis*.

**Supplementary sheet 6.** Relative expression of *GAL3ST1*, *IFT88*, and *TSSK2* in gonad tissues of G0 and G6 generations.

## Data Availability

The review does include data gathering and analysis. Individual genotype data are available on DataDryad (https://datadryad.org/stash/share/‐2k6qUPVI1_0XGddWrQJG4gemda275vhrxO‐1jy2I7o). Benefits from this research accrue from the sharing of our views on the genetic basis for *M. lateralis* to adapt to laboratory culture during breeding and the potential of using this species to explore adaptive evolution of bivalve.
