# Supplementary figures and images for: Signatures of selection in Mulinia lateralis underpinning its rapid adaptation to laboratory conditions
Source: Evol Appl. 2024 Feb 14;17(2):e13657. doi: 10.1111/eva.13657 (PMC10866071; doi:10.1111/eva.13657)

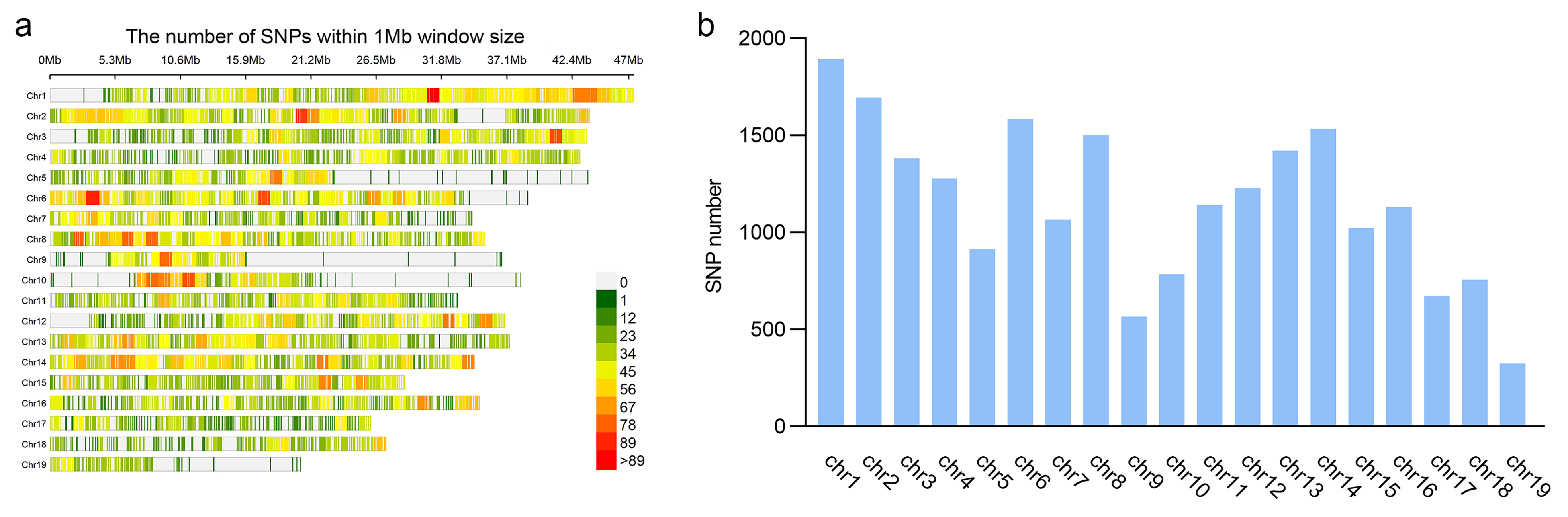

Supplement: Supplementary file 1 — Figure S1. Distribution of single nucleotide polymorphisms (SNPs) in the genome of Mulinia lateralis. (a) Locations and density of SNPs; (b) Numbers of SNPs in different chromosomes. [file EVA-17-e13657-s003.tif]

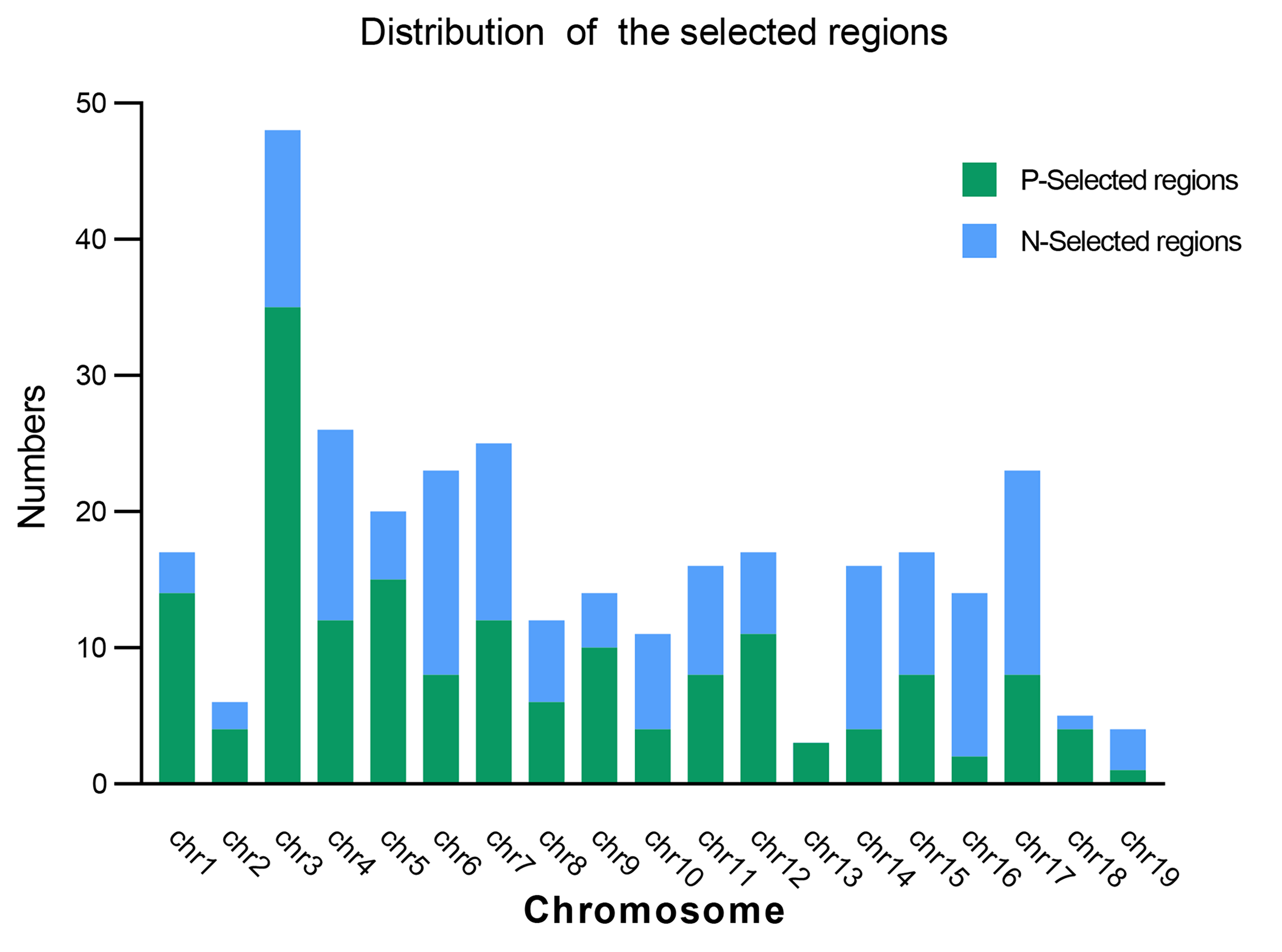

Supplement: Supplementary file 2 — Figure S2. Distribution of selected regions in Mulinia lateralis. The green represents positively selected regions, while the blue represents negatively selected regions. [file EVA-17-e13657-s002.tif]

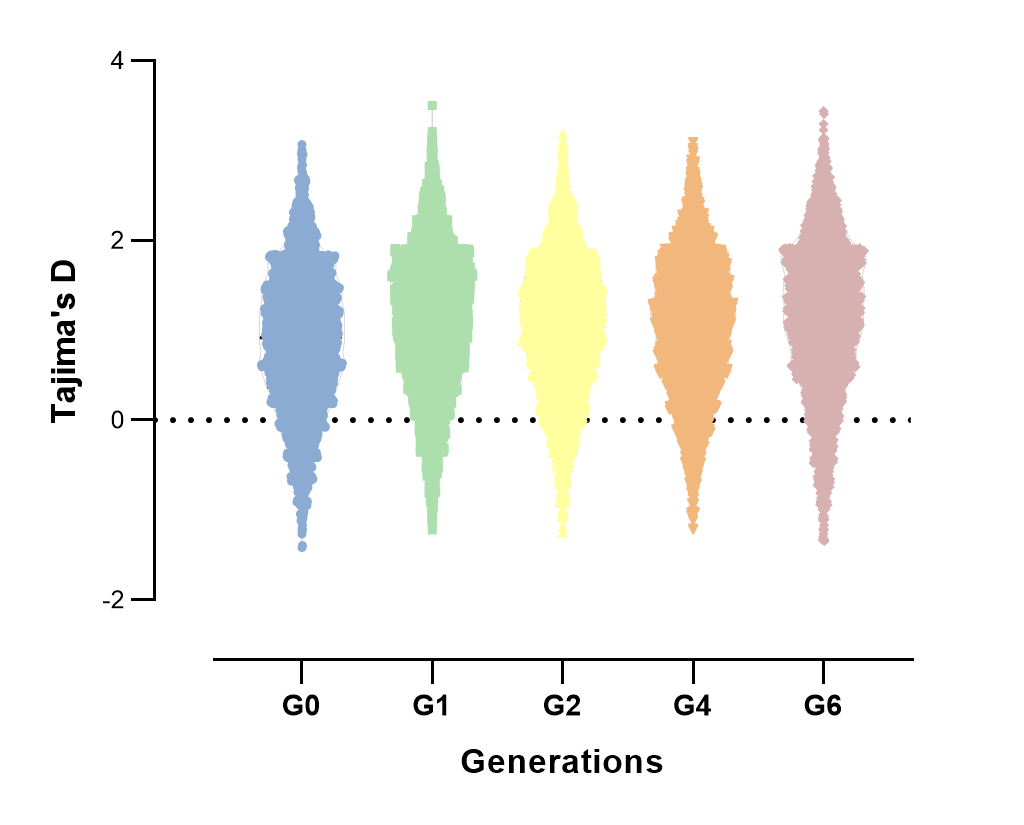

Supplement: Supplementary file 3 — Figure S3. Tajima’s D values in Mulinia lateralis of different generations. [file EVA-17-e13657-s010.tif]

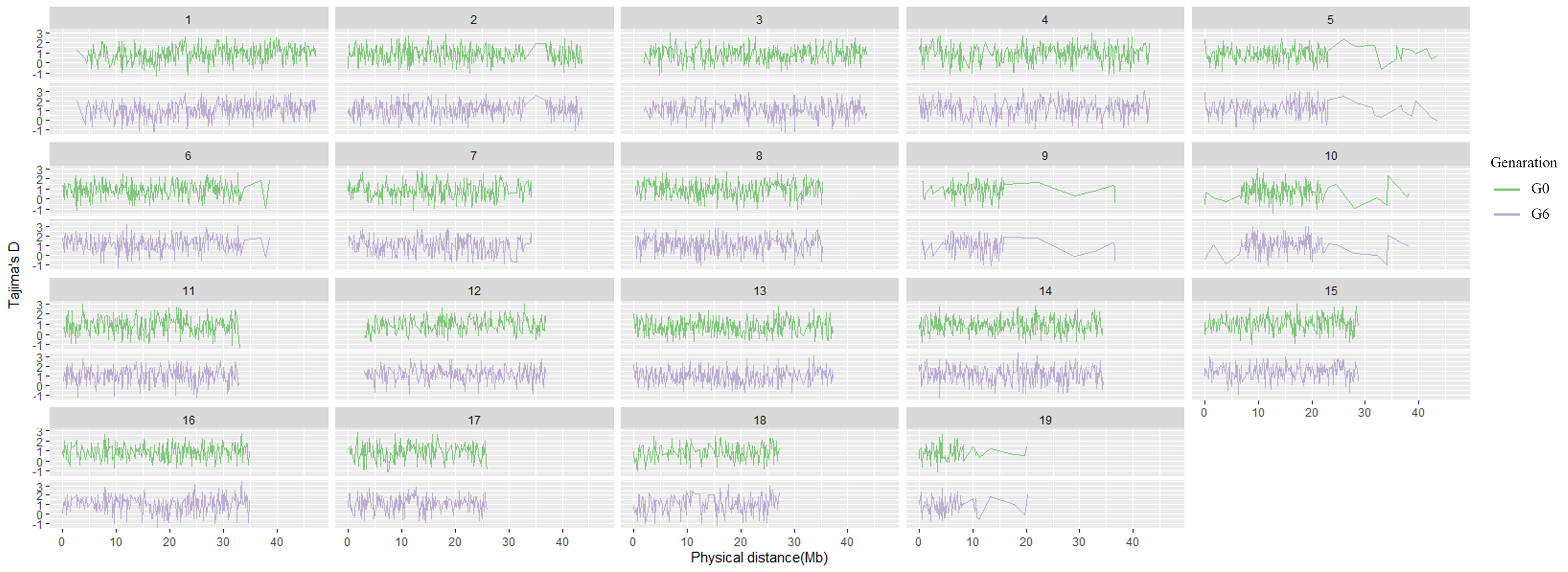

Supplement: Supplementary file 4 — Figure S4. Distribution of Tajima’s D values in genomic regions of Mulinia lateralis. The green represents the G0 generation, while the purple represents the G6 generation. [file EVA-17-e13657-s009.tif]
