# Supplementary material for: Signatures of selection in Mulinia lateralis underpinning its rapid adaptation to laboratory conditions
Source: Evol Appl. 2024 Feb 14;17(2):e13657. doi: 10.1111/eva.13657 (PMC10866071; doi:10.1111/eva.13657)
Supplement: Supplementary file 5 — Table S1. Characteristics and numbers of SNPs in Mulinia lateralis populations. [file EVA-17-e13657-s001.docx]

Table S1. Characteristics and numbers of SNPs in *Mulinia lateralis* populations.

| Category | Type | Counts | Percent (%) |
| --- | --- | --- | --- |
| Region | Upstream | 2,956 | 13.32 |
|  | Downstream | 2,947 | 13.28 |
|  | UTR_5_prime | 49 | 0.22 |
|  | UTR_3_prime | 87 | 0.39 |
|  | Exon | 5,420 | 24.42 |
|  | Intron | 2,399 | 10.81 |
|  | Intergenic | 8,053 | 36.28 |
|  | Splice_site_acceptor | 38 | 0.17 |
|  | Splice_site_donor | 37 | 0.17 |
|  | Splice_site_region | 210 | 0.95 |
