# Supplementary material for: Signatures of selection in Mulinia lateralis underpinning its rapid adaptation to laboratory conditions
Source: Evol Appl. 2024 Feb 14;17(2):e13657. doi: 10.1111/eva.13657 (PMC10866071; doi:10.1111/eva.13657)
Supplement: Supplementary file 6 — Table S2. Summary of nucleotide substitutions in Mulinia lateralis populations. [file EVA-17-e13657-s008.docx]

Table S2. Summary of nucleotide substitutions in *Mulinia lateralis* populations.

|  | A | C | G | T |
| --- | --- | --- | --- | --- |
| A | - | 937 | 2,658 | 2,333 |
| C | 1,186 | - | 473 | 3,520 |
| G | 3,461 | 467 | - | 1,165 |
| T | 2,323 | 2,671 | 1,002 | - |
